# Qingxin Kaiqiao Recipe Improves Cognitive Performance, Inhibits Apoptosis, and Reduces Pathological Deposits in APP/PS1 Double Transgenic Mice via the PI3K/Akt Pathway

**DOI:** 10.1155/2020/3019674

**Published:** 2020-04-28

**Authors:** Shi-Yi Lin, Tian-Qi Wang, Lu-Ting Xu, Xiao-Xiao Lai, Yan Shen, Jian-Wei Lin, Shi-Yu Gao, Hai-Yan Hu

**Affiliations:** ^1^The Second Affiliated Hospital and Yuying Children's Hospital of Wenzhou Medical University, 109 Xue Yuan Xi Road, Lu Cheng District, Wenzhou 325000, China; ^2^The Second Clinical College, Wenzhou Medical University, Wenzhou 325003, China

## Abstract

The traditional Chinese medicine of Qingxin Kaiqiao Recipe (QKR) is effective in the treatment of Alzheimer's disease (AD). This study aims to investigate whether QKR improves the cognitive ability and takes neuroprotective effect on APP/PS1 double transgenic mice via the PI3K/Akt pathway. APP/PS1 double transgenic mice were randomly divided into a model, donepezil-treated, or QKR-treated group (L-QKR: 4.75 mg/kg/d, M-QKR: 9.5 mg/kg/d, and H-QKR: 19 mg/kg/d, respectively). Wild-type C57/BL6J mice were used as the control group. Morris water maze (MWM) was used to test the ability of spatial navigation and memorization; terminal deoxynucleotidyl transferase-mediated dUTP nick end-labelling (TUNEL) assay was applied to test the apoptosis; amyloid protein granule deposition was detected via Methenamine silver staining; Western blot (WB) analysis, immunohistochemistry, and RT-PCR were applied to measure the expression of A*β* and corresponding indicators of the PI3K/Akt pathway. Compared with the model group, QKR significantly relieved the cognitive impairment, reduced the deposition of senile plaques, decreased the expression of GSK-3*α* and A*β*, and increased the expression of p-PI3K, p-Akt, and IDE. In addition, the number of TUNEL-positive cells decreased after treatment using QKR. The current study proved that QKR, especially at the high dose tested, exerted a protective effect on improving learning and memory, inhibiting apoptosis, and reducing the process of pathological degeneration in the hippocampus of AD mice.

## 1. Introduction

Alzheimer's disease (AD), the most common cause of dementia, is characterized by the progressive development of cognitive dysfunction syndrome, including impairment of language, visual space, and memory function to varying degrees [1, 2]. According to a report by the Alzheimer's Disease Association in 2019, with the extension of people's life expectancy, the risk of AD among people over 65 years of age has increased to 1 in 10, and that among people over 85 years old has increased to nearly 1 in 2 [3], which imposes a huge burden on both families and society. The main pathological features of AD currently known are neurofibrillary tangles (NFTs), neuronal death, and senile plaque (SP) [4]. The major component of SP is amyloid-beta (A*β*), whose abnormal aggregation and deposition in the brain can induce a series of neurotoxic effects leading to the occurrence of AD, such as neuronal cell death, synaptic loss, and cognition impairments [5, 6]. Based on the existing studies, the metabolism of A*β* can be influenced by the phosphoinositide 3-kinase (PI3K)/protein kinase B (Akt) pathway [7], which is related to a variety of intracellular regulation and is one of the key pathways for cell cycle regulation and apoptosis [8]. At present, the main methods of treating AD at home and abroad are western medicine and rehabilitation [9]; the former causes undesirable side effects, and the latter is costly and unstable. Given the relative poverty status of the conventional therapy, more and more researchers turn their attention to the traditional Chinese medicine [10, 11]. Qingxin Kaiqiao Recipe (QKR), based on Fumanjian, a notable traditional Chinese medicine compound from Jingyue Quanshu written by Zhang Jingyue in Ming Dynasty, has been widely used in clinic for many years and has produced remarkable effects. QKR is usually used to treat the combination of excess and deficiency syndrome, which can manifest as follows: (1) Qi stagnation (mild disorders of behavior and language, sentimentality, chest oppression, and taut pulse). (2) Yin deficiency (night sweat or spontaneous sweating, dry tongue, and fast pulse). But one who can be treated by QKR needs to have a healthy appetite [9, 12]. QKR is made up of 10 kinds of Chinese herbs, including Radix Rehmanniae (Sheng di huang), Radix Paeoniae Alba (Bai shao), Radix Ophiopogonis (Mai dong), Cortex Moutan Radicis (Mu dan pi), *Poria cocos* (Fu ling), Herba Dendrobii Rhizoma (Shi hu), Rhizoma Acori Tatarinowii (Shi chang pu), Rhizoma Anemarrhenae (Zhi mu), *Sophorae flavescentis* (Ku shen), and Pericarpium Citri Reticulatae (Chen pi). It is currently unknown whether QKR can improve cognitive performance and exert a neuroprotective effect on APP/PS1 double transgenic mice via the PI3K/Akt pathway.

## 2. Materials and Methods

### 2.1. Animals

One hundred male APP/PS1 double transgenic mice of specific pathogen free (SPF) grade (25 ± 2 g), aged 3 months, were purchased from Beijing HFK Co., Ltd. (Beijing, China; certification number SCXK 2014-0004). Twenty male wild-type C57/BL6J mice (25 ± 2 g) were purchased from Shanghai Slake Laboratory Animal Co., Ltd. (Shanghai, China; certification number SCXK 2012-0002). Mice were housed in the animal experimental center of Wenzhou Medical University (Zhejiang, China; certification SYXK number 2016-0006). Enough food and water were accessible to the mice reared in standard laboratory cages with a 12-hour light/dark cycle. All animal experiments were conducted in accordance with the ethical requirements approved by the Chinese Association of Accreditation of Laboratory Animal Care.

### 2.2. Reagents and Antibodies

The 3,3-diaminobenzidine (DAB) reagent kit was obtained from Hubei Zhong Xin Biological Technology Co., Ltd. (Wuhan, China). All primers were obtained from Sunny Biological Science and Technology Co., Ltd. (Shanghai, China). Donepezil was purchased from Eisai Pharmaceutical Co., Ltd. (Suzhou, China) (Number: 100223A). Primary antibodies against PI3K, p-PI3K, Akt, p-Akt, GSK-3*α*, IDE, and A*β* were purchased from Abcam (Cambridge, UK). Primary antibody against *β*-actin was obtained from Bioworld Technology, Inc. (St. Louis Park, MN, USA**).** Horseradish peroxidase (HRP)-conjugated IgG secondary antibody was purchased from Santa Cruz Biotechnology Inc (Delaware, USA), and a BCA protein assay kit was obtained from Beyotime Biotechnology Co., Ltd. (Shanghai, China).

### 2.3. Preparation of QKR

QKR is composed of 10 herbs: Radix Rehmanniae (Sheng di huang), Radix Paeoniae Alba (Bai shao), Radix Ophiopogonis (Mai dong), Cortex Moutan Radicis (Mu dan pi), *Poria Cocos* (Fu ling), Herba Dendrobii Rhizoma (Shi hu), Rhizoma Acori Tatarinowii (Shi chang pu), Rhizoma Anemarrhenae (Zhi mu), *Sophorae flavescentis* (Ku shen), and Pericarpium Citri Reticulatae (Chen pi), in a ratio of 2 : 2 : 2 : 2 : 2 : 2 : 2 : 1.5 : 1.5 : 1 on a dry-weight basis. All herbs were provided by the Second Affiliated Hospital of Wenzhou Medical University and verified by the Department of Chinese Materia Medical of Wenzhou Medical University. To make 1 g/mL drug stocks, the raw herbs were decocted with 10 times the volume of distilled water, extracted twice, filtered, and concentrated, and then, the drug stocks were stored at 4°C until use.

### 2.4. Experimental Schedule

**Figure 1:**
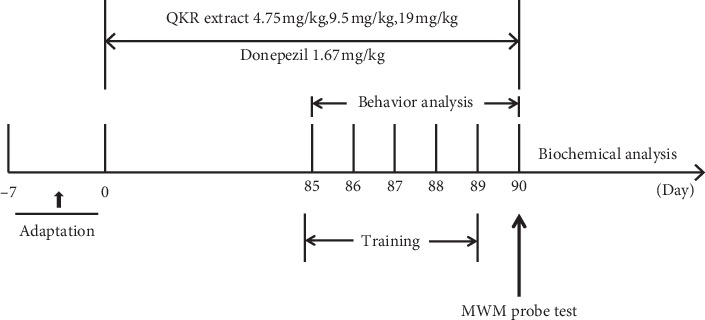
Study on the effect of QKR on APP/PS1 mice by behavioral and biochemical analysis timetable. QKR, Qingxin Kaiqiao Recipe; WMW, Morris water maze.

The 3-month-old APP/PS1 mice were randomly divided into 5 groups by the random number table method (model group, *n* = 20; donepezil group: 1.67 mg/kg/d, *n* = 20; high dose of QKR: 19 mg/kg/d, *n* = 20; medium dose of QKR: 9.5 mg/kg/d, *n* = 20; low dose of QKR: 4.75 mg/kg/d, *n* = 20), and then administered with abovementioned medicines via oral gavage. Wild-type C57/BL6J mice were used as a normal control (*n* = 20) throughout the study and were given the same volume of saline water (0.2 mL/10 g weight). Each group was treated once a day at 10:00 am for a period of 3 months (Figure 1).

### 2.5. Behavioral Test

Spatial learning and memory of mice was assessed in Morris water maze (MWM) as published previously by two investigators completely blind to the treatment of the animals [13]. The 90 cm diameter pool was divided into four equal quadrants (South, East, North, and West), and it was filled with water maintained at 24°C with a hidden platform (10 cm diameter and 1.5 cm below the surface of water) located in the middle of the third quadrant. The test was performed for 6 days, once per day. In the first five days, each mouse was placed randomly into the water at one of the four quadrants, facing the wall of the pool. The route of navigation was recorded by the camera above the pool and analysed by the MWM system. When finding the platform within 60 s, the mouse was allowed to stand on the platform for 10 s. If not, it was placed on the platform by investigators for 10 s. The 6th day was for spatial probe, investigators removed the platform and placed the mice into the pool to swim freely for 60 s. The number of times they crossed the original platform within 60 s was recorded by the camera.

### 2.6. Hippocampus Collection

In order to conduct different experiments, we divided mice in each group into two parts after the MWM test. Five mice selected at random in each group were anesthetized with an intraperitoneal injection of 10% chloral hydrate; the brains were removed quickly and placed in 4% paraformaldehyde, and the tissues were fixed in the 4% paraformaldehyde for 24 h, and then, dehydrated by passage through a gradient alcohol dehydration at room temperature. The tissues, transparentized with xylene, were then soaked in wax, embedded in paraffin, and finally, cut into sections of 5 *μ*m thick. The rest of mice (*n* = 15/group) were killed after anaesthesia; the hippocampus was dissected under a dissecting microscope, placed in the EP tube, and stored at −80°C refrigerator.

### 2.7. TUNEL Staining

TUNEL technique was performed according to the manufacturer's protocol supplied within the TUNEL pod kit (Roche, USA). The dewaxed slices were placed into 3% hydrogen peroxide in methanol for 15 min and were added 100 *μ*l protease K (20 *μ*g/ml) for 20 min. After washing with PBS, TUNEL reaction solution was added to the slices at 37°C for 1 h, and then, the POD was added to the slices. Finally, the slices were rinsed 3 times and stained with DAB and hematoxylin. The number of viable TUNEL-positive neurons in the hippocampus was observed by an Olympus fluorescence positive microscope.

### 2.8. Methenamine Silver Staining

After the slices were conventionally dewaxed, 0.5% periodate and 8% chromic acid were added for 15 min and 60 min, respectively. Then, the slices were washed with distilled water for 5 min, placed into preheated methenamine silver (Leagene, Beijing), and kept in an incubator at 60°C for 45 min. Finally, the slices were washed and added 1% gold chloride aqueous solution to tint for 2 min. The positive granules were calculated in Image-Pro Plus 6.0 (IPP 6.0).

### 2.9. Western Blot Analysis

The frozen hippocampal tissues were taken out from −80°C refrigerator and lysed in RIPA lysis buffer containing a protease inhibitor cocktail. After calculated by the BCA protein assay kit, the total protein was separated by 6% or 8% SDS-PAGE and then transferred to polyvinylidene difluoride (PVDF) membranes. The membranes were blocked in a skim milk blocking buffer for 2 h, washed 3 times with TBS/0.1% Tween 20, and incubated overnight with the following primary antibodies: PI3K (1 : 1000), p-PI3K (1 : 1000), Akt (1 : 1000), p-Akt (1 : 1000), GSK-3*α* (1 : 1000), IDE (1 : 1000), A*β* (1 : 1000), and *β*-actin (1 : 1000) at 4°C. The membranes were then washed 3 times with TBS/0.1% Tween 20 and incubated with HRP-conjugated IgG secondary antibody (1 : 1000) for 2 h at room temperature. The bands were detected by the chemiluminescence reagents ECL Western blotting detection reagents (USA) and finally analysed by AlphaEase FC gel image analysis software.

### 2.10. Immunohistochemistry

The fixed tissues were removed from refrigerator, dehydrated with a graded alcohol, impregnated in xylene, embedded in paraffin, and cut into slices of 5 *μ*m thick. Then, 3% H_2_O_2_ was used to close endogenous peroxidase. After closuring with 10% goat serum for 10 min at room temperature, the slices were incubated with primary antibodies against PI3K (1 : 100), p-PI3K (1 : 100), Akt (1 : 100), p-Akt (1 : 100), and A*β* (1 : 100) overnight at 4°C. Lastly, positive granules were detected by Polink-2 plus ®Polymer HRP Detection System. The optical density (OD) (IOD/area) was calculated in Image-Pro Plus (IPP) 6.0.

### 2.11. Quantitative Real-Time PCR

Total RNA from hippocampus was isolated from TRIzol reagent according to the manufacturer's procedures. The absorbance values of RNA at OD 260 and OD 280 as well as the RNA content were detected by an M200 pro full-wave length multifunction microplate reader (Tecan, Switzerland). The cDNA was synthesized with reverse transcriptase (Thermo, USA). The quantitative polymerase chain reaction (qPCR), which includes 0.5 *μ*l of each primer, 1 *μ*l of the cDNA sample, 5 *μ*l of the SYBR Green I Master, and 3 *μ*l of the DEPC water, were performed in LightCycler® 480II/96 (Roche, Switzerland). The reaction was set as following: 95°C for 4 min, followed by 35 cycles of 95°C for 10 s, 54°C for 10 s, and 72°C for 10 s, the signal was detected at 72°C, and each PCR reaction was performed in duplicate. Finally, the results were analysed by the 2^−△△Ct^ method (Table 1).

### 2.12. Statistical Analysis

All data were processed using SPSS 18.0 statistical software and presented as the mean ± standard deviation. The data of each group were compared by the independent samples *t*-test and one-way ANOVA. Intergroup comparison was performed by the LSD method. In all analyses, *P* < 0.05 was taken to indicate statistical significance.

## 3. Results

### 3.1. Behavioral Test Results

**Figure 2:**
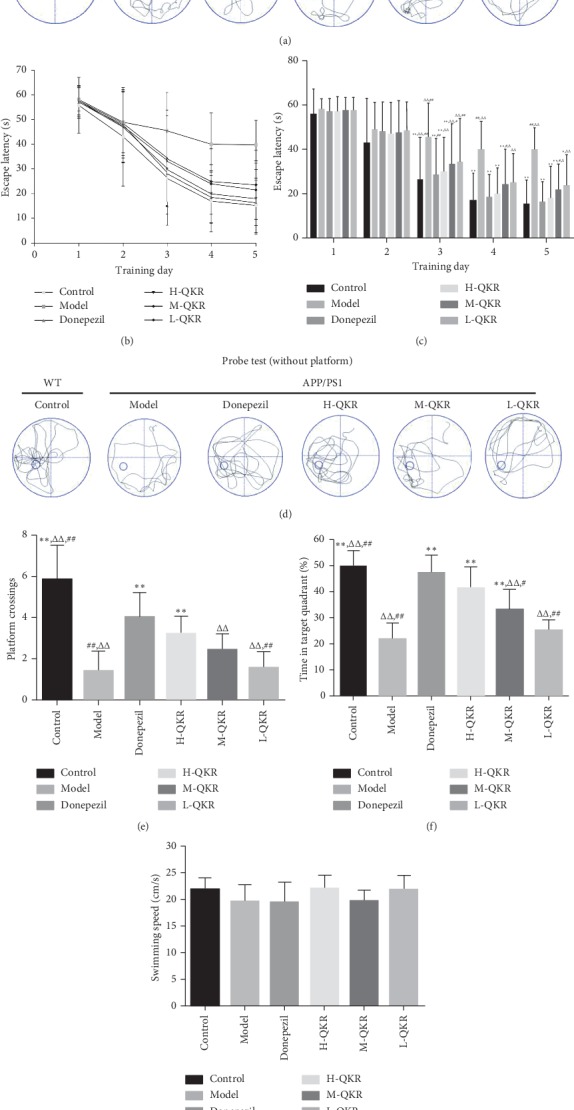
QKR improves the spatial and long-term memory decline in APP/PS1 mice. (a) Typical path tracking in hidden platform navigation experiment. (b) Average latencies curve of one trial a day in the five consecutive days. (c) Quantitative analysis of escape latency in each group. (d) Representative path tracking in the probe tests without hidden platform. (e) The average number of times the mice crossed the platform in 60 seconds. (f) The percentage of searching time that the mice of individual groups spent in the target quadrant. (g) Swimming speed of mice in each group. Compared with the model group, ^*∗*^*P* < 0.05, ^*∗∗*^*P* < 0.01; compared with the donepezil group, ^△^*P* < 0.05, ^△△^*P* < 0.01; compared with the H-QKR group, ^#^*P* < 0.05, ^##^*P* < 0.01. L-QKR, low-dose QKR group; M-QKR, medium-dose QKR group; H-QKR, high-dose QKR group. QKR, Qingxin Kaiqiao Recipe; WT, wild type.

In the MWM test, compared with the control group, the path tracking of APP/PS1 mice was disorganized. The QKR-treated group, especially the group of H-QKR, showed a selective search way for the platform and shorter path length than the model group did (Figure 2(a)). The average escape latency of 5 consecutive days of each group was displayed in curves and histogram (Figures 2(b) and 2(c)). There was no clear distinction among different groups in the first two days. However, from the third day onwards, the differences became more and more obvious. The model group maintained a longest escape route from start to finish, while the control group showed the shortest one on each day. The latency in the donepezil and H-QKR groups presented significant differences compared with that in the model group from the 3rd to 5th day (*P* < 0.01).

The above results of the navigation tests with hidden platform were supported by a subsequent probe trial without the platform. The typical path tracking of each group within 60 s was shown in Figure 2(d). Within the similar total path length, the model group swam randomly throughout the tank. Compared with the model group, the H-QKR group and donepezil groups had more cross-platform locations and higher percentage of target quadrant searching time (*P* < 0.01) (Figures 2(e) and 2(f)). These results provided evidence on the significant compensating effect of H-QKR on cognitive deficits. Furthermore, to exclude the possibility that the above results were due to visual or motor impairment, the swimming speed of mice was measured using a visible platform after the probe test. As shown in Figure 2(g), there were no statistically significant differences in average swimming speed among groups (*P* > 0.05).

### 3.2. TUNEL Staining Results

**Figure 3:**
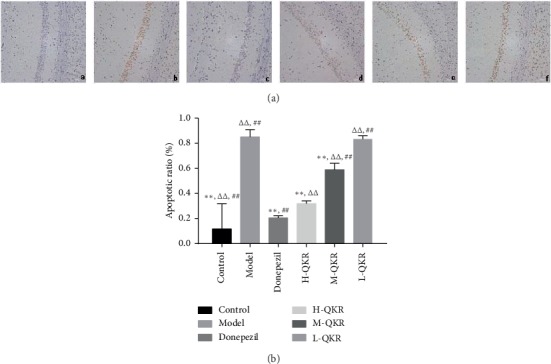
Effects of QKR on the number of apoptotic cells. (a) TUNEL staining in the hippocampal CA1 area of each group of mice (original magnification, ×200). (b) Statistical analysis results of TUNEL staining. (a–f) Control, model, donepezil, H-QKR, M-QKR, and L-QKR group, respectively. Compared with the model group, ^*∗∗*^*P* < 0.01; compared with the donepezil group, ^△△^*P* < 0.01; compared with the H-QKR group, ^##^*P* < 0.01. L-QKR, low-dose QKR group; M-QKR, medium-dose QKR group; H-QKR, high-dose QKR group. QKR, Qingxin Kaiqiao Recipe; TUNEL, terminal deoxynucleotidyl transferase-mediated dUTP nick end-labelling.

The apoptotic cells stained by TUNEL staining were brown, the nucleus was blue, and the background was light blue. The results showed that the apoptosis in the model group was quite obvious, compared with other groups (*P* < 0.01), but the situation got better after treatment with donepezil or QKR, especially the donepezil group, which showed the most palpable effect in the treatment groups (*P* < 0.01). In addition, H-QKR produced the strongest effect among the three doses of QKR groups (*P* < 0.01) (Figure 3).

### 3.3. Methenamine Silver Granule Staining Results

**Figure 4:**
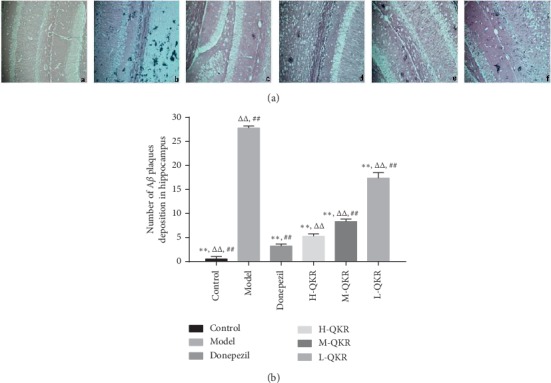
Methenamine silver granule staining results. (a) Methenamine silver granule staining in the hippocampal CA1 area of each group of mice (original magnification, ×200). (b) Statistical analysis results of plaques. (a–f) Control, model, donepezil, H-QKR, M-QKR, and L-QKR group, respectively. Compared with the model group, ^*∗∗*^*P* < 0.01; compared with the donepezil group, ^△△^*P* < 0.01; compared with the H-QKR group, ^##^*P* < 0.01. L-QKR, low-dose QKR group; M-QKR, medium-dose QKR group; H-QKR, high-dose QKR group. QKR, Qingxin Kaiqiao Recipe.

Senile plaques were black with a light brown background. Compared with other groups, the number of positive plaques in the hippocampus of mice in the model group increased significantly (*P* < 0.01). After treatment with donepezil or QKR, the number of positive plaques became fewer; however, the effect of the donepezil group was better than that of QKR groups (*P* < 0.01). There was also a significant difference in the number of senile plaques between the high dose of the QKR group and the medium and low dose of QKR groups (*P* < 0.01) (Figure 4).

### 3.4. Protein Levels of PI3K, p-PI3K, Akt, p-Akt, GSK-3*α*, IDE, and A*β* in the Hippocampus

**Figure 5:**
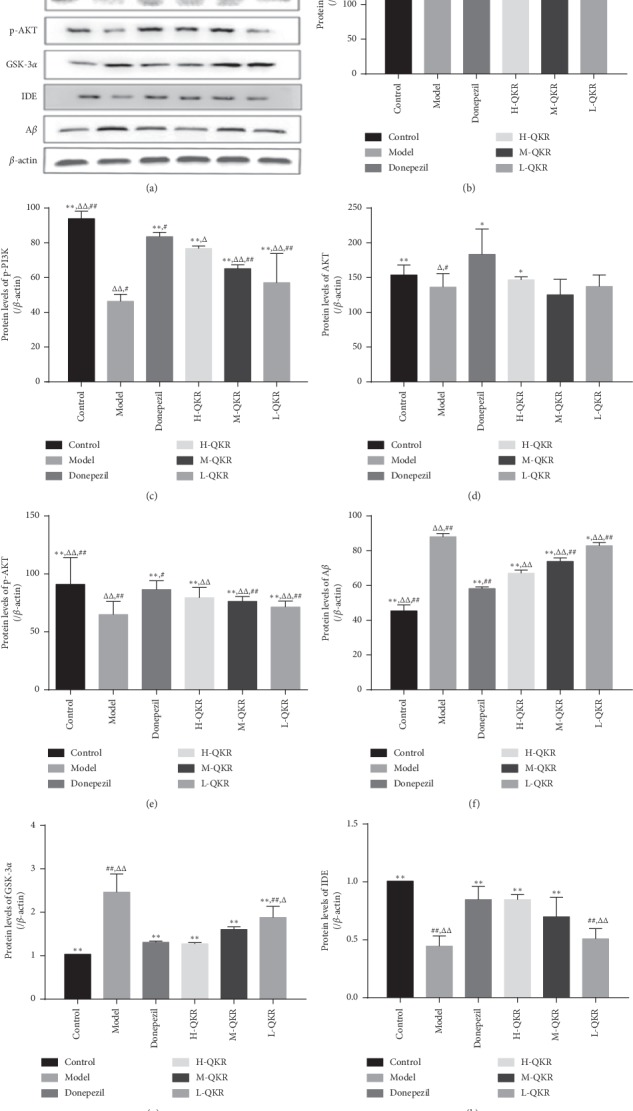
Protein expression of PI3K, p-PI3K, Akt, p-Akt, GSK-3*α*, IDE, and A*β* in the hippocampus. (a) WB for PI3K, p-PI3K, Akt, p-Akt, GSK-3*α*, IDE, and A*β* expression in the hippocampus of mice. (b–h) ODs indicative of PI3K, p-PI3K, Akt, p-Akt, A*β*, GSK-3*α*, and IDE protein expression. Compared with the model group, ^*∗*^*P* < 0.05, ^*∗∗*^*P* < 0.01; compared with the donepezil group, ^△^*P* < 0.05, ^△△^*P* < 0.01; compared with the H-QKR group, ^#^*P* < 0.05, ^##^*P* < 0.01. L-QKR, low-dose QKR group; M-QKR, medium-dose QKR group; H-QKR, high-dose QKR group. QKR, Qingxin Kaiqiao Recipe; WB, western blot.

The expression levels of A*β* and GSK-3*α* protein in the model group were higher than that in other groups (*P* < 0.05, *P* < 0.01), while the levels of p-PI3K, p-Akt, and IDE were lower than that in the remaining groups (*P* < 0.01). The dose of 19 mg/kg/d QKR group produced the strongest effect among the three doses of QKR groups, which exhibited the highest expression levels of p-PI3K, p-AKT, and IDE protein (*P* < 0.01) and the lowest expression levels of GSK-3*α* and A*β* protein (*P* < 0.01); however, there were no evident differences in the expression of PI3K and Akt protein among QKR groups (*P* > 0.05) (Figure 5).

### 3.5. Immunohistochemistry Results

**Figure 6:**
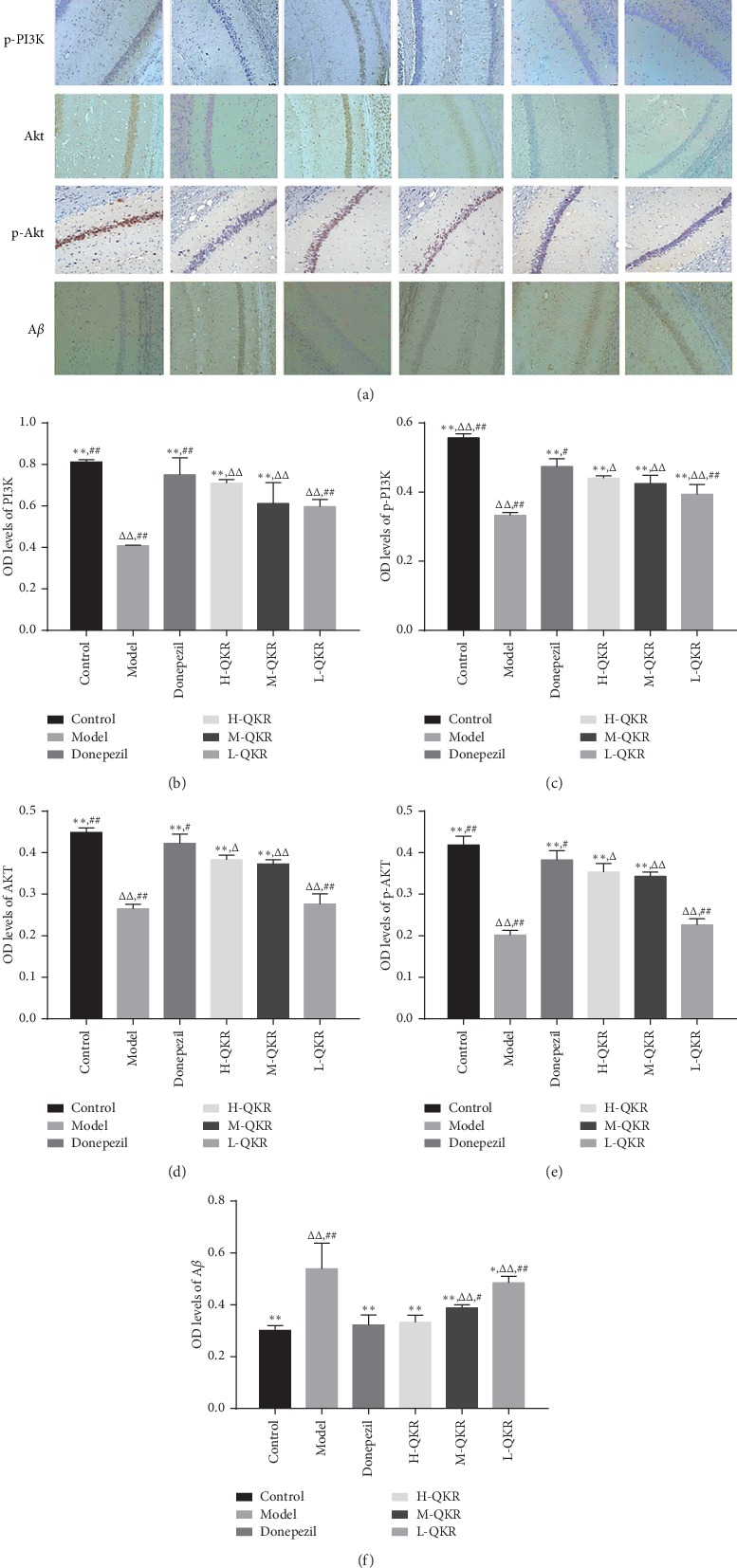
Immunohistochemistry for the protein expression in the hippocampus of APP/PS1 mice. (a) Immunohistochemistry for PI3K, p-PI3K, Akt, p-Akt, and A*β* expression in the CA1 region of the hippocampus for each group (original magnification ×200). (b–f) Analysis of PI3K, p-PI3K, Akt, p-Akt, and A*β* levels by immunohistochemical staining. Compared with the model group, ^*∗*^*P* < 0.05, ^*∗∗*^*P* < 0.01; compared with the donepezil group, ^△^*P* < 0.05, ^△△^*P* < 0.01; compared with the H-QKR group, ^#^*P* < 0.05, ^#^*P* < 0.05. L-QKR, low-dose QKR group; M-QKR, medium-dose QKR group; H-QKR, high-dose QKR group. QKR, Qingxin Kaiqiao Recipe.

After immunohistochemical staining, the positive cells were stained brown with a light blue or brown-grey background. The results showed that the expression levels of A*β* in the model group were significantly higher than that in other groups, and the expression levels of PI3K, p-PI3K, AKT, and p-AKT in the model group was much lower than the remaining groups (*P* < 0.05,  *P* < 0.01). Although the medication group did not achieve the effect of the normal group, the results were improved after treatment with donepezil or QKR. Among them, the donepezil group was the most effective one, followed by the H-QKR group (Figure 6).

### 3.6. Gene Expression of PI3K, Akt, and A*β* in the Hippocampus

**Figure 7:**
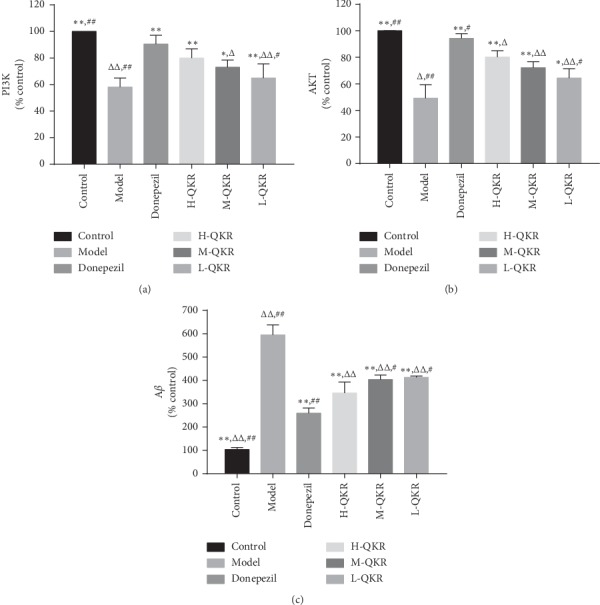
Gene expression of PI3K, AKT, and A*β* in the hippocampus. Compared with the model group, ^*∗*^*P* < 0.05, ^*∗∗*^*P* < 0.01; compared with the donepezil group, ^△^*P* < 0.05, ^△△^*P* < 0.01; compared with the H-QKR group, ^#^*P* < 0.05, ^##^*P* < 0.01. L-QKR, low-dose QKR group; M-QKR, medium-dose QKR group; H-QKR, high-dose QKR group. QKR, Qingxin Kaiqiao Recipe.

The expression levels of A*β* in the model group was the highest among all groups (*P* < 0.05,  *P* < 0.01); meanwhile, the model group showed the lowest expression levels of PI3K and Akt (*P* < 0.05,  *P* < 0.01). After treatment with QKR or donepezil, the expression of A*β* decreased to varying degrees, and the expression of PI3K and Akt increased to different levels (Figure 7).

## 4. Discussion

As a major component of senile plaques, A*β* peptide causes neurotoxicity and plays a key role in the pathogenesis of AD [14, 15]. The accumulation of A*β* can activate apoptosis and result in neuron cell death [16]. It was demonstrated that the level of A*β* is increased in AD brains and is related to the severity of the disease [17]. A classical AD model, APP/PS1 double transgenic mice were used in this research. The structure of the mice was based on A*β* pathology, with learning and memory impairment and detectable A*β* in the brain at 4 months old and significant amyloidosis at 6 months of age. And three-month-old mice usually have shown mild cognitive impairment accompanied by mild A*β* deposition [18, 19]. From the age of 3 months, the mice were treated with diluted QKR extract for 3 months. It was exciting to see from the results of MWM and silver granule staining that after 3 months of QKR application, the cognitive performance of QKR-treated AD mice was improved compared with the model group, and the number of senile plaques was significantly reduced, indicating its role in improving cognition ability and reducing A*β* deposition.

QKR is a formula based on Fu Man Jian, a famous decoction composed of 10 traditional Chinese herbs. The major effective chemical compounds are summarized as follows: (1) Rhizoma Acori Tatarinowii (Shi chang pu): asarone is one of the major ingredients of Rhizoma Acori Tatarinowii. Asarone and levodopa coadministration increase the striatal dopamine level in 6-hydroxydopamine-induced rats by modulating P-glycoprotein and tight junction proteins at the blood-brain barrier (BBB) and promoting levodopa into the brain [20]. (2) Radix Ophiopogonis (Mai dong): Methylophiopogonanone A (MO-A), an active homoisoflavonoid of Radix Ophiopogonis which can markedly attenuate BBB damage in vitro [21]. (3) Radix Ophiopogonis (Mai dong): ruscogenin, an important steroid sapogenin derived from Radix Ophiopogonis, which can attenuate cerebral ischemia-induced BBB dysfunction [22]. (4) Cortex Moutan Radicis (Mu dan pi): Paeonol (2′-hydroxy-4′-methoxyacetophenone), a natural component and principle bioactive compound isolated from the root bark of Cortex Moutan Radicis, is actively transported from blood to the brain across the BBB by a carrier-mediated transporter system [23]. (5) Rhizoma Anemarrhenae (Zhi mu): compound: 2,6,4′-trihydroxy-4-methoxybenzophenone in Rhizoma Anemarrhenae (Zhi mu) was found to be an antioxidant with very high BBB permeability [24]. Therefore, the effect of QKR may be related to its effective chemical components which can penetrate the blood-brain barrier and finally enter the brain. Our previous in vivo experiments have proved that QKR could exert neuroprotection in AD mice by inhibiting apoptosis via multiple pathways. QKR could affect apoptosis-related protein expression, such as stimulation of antiapoptotic protein regulator Bcl-2 and inhibition of proapoptotic protein regulator Bax and caspase-3 [25]. Existing studies have reported that the PI3K/Akt pathway is involved in the activity of different kinds of cells, including the hippocampal neuronal cells [26, 27]. Akt is a crucial downstream effector of phosphatidylinositol 3-kinase (PI3K), and its phosphorylation is essential in cell proliferation and cell death [28]. When the Akt is activated by the PI3K pathway, it can lead to phosphorylation of glycogen synthesis kinase-3*α* (GSK-3*α*), thus inhibiting the activity of GSK-3*α* and then suppressing the formation of A*β* by interfering with the activity of secretase [29, 30]. Besides, the PI3K/Akt pathway may also be involved in the regulation of IDE activity, an important A*β* degrading enzyme, thereby affecting the degradation of extracellular A*β* [31, 32]. It could be seen from the results of WB analysis that QKR-treated AD mice showed higher levels of p-PI3K, p-Akt, and IDE and lower levels of GSK-3*α* and A*β* than the model group. The results of immunohistochemistry and RT-PCR showed that the QKR-treated AD mice showed higher levels of p-PI3K and p-Akt and lower levels of A*β* than the model group, indicating the role of QKR in increasing the activity of the PI3K/Akt pathway. Furthermore, the TUNEL results revealed that after QKR treatment, there were fewer apoptotic cells in the AD mice, which unfolded an antiapoptotic role of QKR.

To sum up, QKR has a good effect on treating AD. The present experiment showed that the 19 mg/kg/d QKR (H-QKR) group was the most effective one among the three doses of QKR groups. However, whether there exists a higher dose of QKR that produce more effective role remains to be further explored. The effect of QKR may be related to its effective chemical components which can penetrate the blood-brain barrier and finally enter the brain. We will perform HPLC-fingerprint analysis in our next experiment to explore the mechanism of QKR. Moreover, an inhibitor of PI3K/Akt pathway needs to be applied in the future to better prove that QKR can indeed work through this pathway.

## 5. Conclusion

QKR may slow down the progress of AD by alleviating the level of A*β*, improving the cognitive function, and inhibiting apoptosis in the APP/PS1 double transgenic mice via the PI3K/Akt pathway, especially the high dose group of 19 mg/kg/d, which showed almost similar effects with the positive control group of donepezil, an drug with clinical validity certification.

## Figures and Tables

**Table 1:** Primer sequences.

Primers		Sequence (5′-3′)
PI3K	Forward	GGAACCAACTTCAGCAGCTC
	Reverse	GAGCCTCTCTGTCCTGTTGG

Akt	Forward	CCGCCTGATCAAGTTCTCCT
	Reverse	TTCAGATGATCCATGCGGGG

A*β*	Forward	CTGGAGGTGCCCACTGATG
	Reverse	GGGTCTGACTCCCATTTTCC

*β*-Actin	Forward	CGTTGACATCCGTAAAGACCTC
	Reverse	TAGGAGCCAGGGCAGTAATCT

## Data Availability

The basic studying data used to support the findings of this study were supplied by Dr. Tianqi Wang under license and so cannot be made freely available. Requests for access to these data should be made to Dr. Tianqi Wang (tenkiou@foxmail.com).

## References

[1] Philippens I. H., Ormel P. R., Baarends G., Johansson M., Remarque E. J., Doverskog M. (2017). Acceleration of amyloidosis by inflammation in the amyloid-beta marmoset monkey model of Alzheimer’s disease. *Journal of Alzheimer’s Disease*.

[2] Yin S., Ran Q., Yang J., Zhao Y., Li C. (2020). Nootropic effect of neferine on aluminium chloride-induced Alzheimer’s disease in experimental models. *Journal of Biochemical and Molecular Toxicology*.

[3] Ryu J. C., Zimmer E. R., Rosa-Neto P., Yoon S. O. (2019). Consequences of metabolic disruption in Alzheimer’s disease pathology. *Neurotherapeutics: The Journal of the American Society for Experimental NeuroTherapeutics*.

[4] Sun X., Nie B., Zhao S. (2020). Distinct relationships of amyloid-beta and tau deposition to cerebral glucose metabolic networks in Alzheimer’s disease. *Neuroscience Letters*.

[5] Sarkar S., Engler-Chiurazzi E. B., Cavendish J. Z. (2019). Over-expression of miR-34a induces rapid cognitive impairment and Alzheimer’s disease-like pathology. *Brain Research*.

[6] Yang T., Zhu Z., Yin E. (2019). Alleviation of symptoms of Alzheimer’s disease by diminishing A*β* neurotoxicity and neuroinflammation. *Chemical Science*.

[7] Kim B., Feldman E. L. (2012). Insulin resistance in the nervous system. *Trends in Endocrinology and Metabolism*.

[8] Yang S. X., Polley E., Lipkowitz S. (2016). New insights on PI3K/AKT pathway alterations and clinical outcomes in breast cancer. *Cancer Treatment Reviews*.

[9] Hu H. Y., Cui Z. H., Li H. Q. (2014). Fumanjian, a classic Chinese herbal formula, can ameliorate the impairment of spatial learning and memory through apoptotic signaling pathway in the hippocampus of rats with A*β* 1-40-induced Alzheimer’s disease. *Evidence-Based Complementary and Alternative Medicine*.

[10] Cai H., Luo Y., Yan X. (2018). The mechanisms of bushen-yizhi formula as a therapeutic agent against Alzheimer’s disease. *Scientific Reports*.

[11] Hu Y. R., Xing S. L., Chen C., Shen D. Z., Chen J. L. (2019). Tiaoxin recipe, a Chinese herbal formula, inhibits microRNA-34a expression in the APPswe/PS1ΔE9 mouse model of Alzheimer’s disease. *Journal of Integrative Medicine*.

[12] Fu C., Zhang N. L., Chen B. X. (2017). Identification and classification of traditional Chinese medicine syndrome types among senior patients with vascular mild cognitive impairment using latent tree analysis. *Journal of Integrative Medicine*.

[13] Wang S., Jiang W., Ouyang T. (2019). Jatrorrhizine balances the gut microbiota and reverses learning and memory deficits in APP/PS1 transgenic mice. *Scientific Reports*.

[14] Lin M. W., Chen Y. H., Yang H. B., Lin C. C., Hung S. Y. (2019). Galantamine inhibits A*β*-induced neurotoxicity by enhancing *α*7nAChR expression as a cargo carrier for LC3 binding and A*β* engulfment during autophagic degradation. *Neurotherapeutics*.

[15] Deshpande A., Mina E., Glabe C., Busciglio J. (2006). Different conformations of amyloid beta induce neurotoxicity by distinct mechanisms in human cortical neurons. *Journal of Neuroscience*.

[16] Zhang R., Zheng Y., Hu F. (2020). Effect of (m)VD-hemopressin against A*β*1-42-induced oxidative stress and apoptosis in mouse hippocampal neurons. *Peptides*.

[17] McLean C. A., Cherny R. A., Fraser F. W. (1999). Soluble pool of Abeta amyloid as a determinant of severity of neurodegeneration in Alzheimer’s disease. *Annals of Neurology*.

[18] Xiao S., Song L. L., Li J. T. (2020). Intraperitoneal administration of monoclonal antibody against pathologic A*β*42 aggregates alleviated cognitive deficits and synaptic lesions in APP/PS1 mice. *Journal of Alzheimer’s Disease*.

[19] Li Z., Zhang X. B., Gu J. H., Zeng Y. Q., Li J. T. (2020). Breviscapine exerts neuroprotective effects through multiple mechanisms in APP/PS1 transgenic mice. *Molecular and Cellular Biochemistry*.

[20] Huang L., Deng M., He Y., Lu S., Ma R., Fang Y. (2016). *β*-asarone and levodopa co-administration increase striatal dopamine level in 6-hydroxydopamine induced rats by modulating P-glycoprotein and tight junction proteins at the blood-brain barrier and promoting levodopa into the brain. *Clinical and Experimental Pharmacology & Physiology*.

[21] Lin M., Sun W., Gong W., Zhou Z., Ding Y., Hou Q. (2015). Methylophiopogonanone A protects against cerebral ischemia/reperfusion injury and attenuates blood-brain barrier disruption in vitro. *PLoS One*.

[22] Cao G., Jiang N., Hu Y. (2016). Ruscogenin attenuates cerebral ischemia-induced blood-brain barrier dysfunction by suppressing TXNIP/NLRP3 inflammasome activation and the MAPK pathway. *International Journal of Molecular Sciences*.

[23] Gyawali A., Krol S., Kang Y. S. (2019). Involvement of a novel organic cation transporter in Paeonol transport across the blood-brain barrier. *Biomolecules & Therapeutics*.

[24] Zhu J., Yi X., Zhang J., Chen S., Wu Y. (2018). Rapid screening of brain-penetrable antioxidants from natural products by blood-brain barrier specific permeability assay combined with DPPH recognition. *Journal of Pharmaceutical and Biomedical Analysis*.

[25] Gao S., Lin J., Wang T. (2019). Qingxin kaiqiao fang ameliorates memory impairment and inhibits apoptosis in APP/PS1 double transgenic mice through the MAPK pathway. *Drug Design, Development and Therapy*.

[26] Hu Y., Chen W., Wu L. (2019). TGF-*β*1 restores hippocampal synaptic plasticity and memory in alzheimer model via the PI3K/Akt/Wnt/*β*-Catenin signaling pathway. *Journal of Molecular Neuroscience*.

[27] Ren Z., Yang M., Guan Z., Yu W. (2019). Astrocytic *α*7 nicotinic receptor activation inhibits amyloid-*β* aggregation by upregulating endogenous *α*B-crystallin through the PI3K/Akt signaling pathway. *Current Alzheimer Research*.

[28] Yang J., Zhu X. J., Jin M. Z., Cao Z. W., Ren Y. Y., Gu Z. W. (2020). Osthole induces cell cycle arrest and apoptosis in head and neck squamous cell carcinoma by suppressing the PI3K/AKT signaling pathway. *Chemico-Biological Interactions*.

[29] de la Monte S. M., Wands J. R. (2005). Review of insulin and insulin-like growth factor expression, signaling, and malfunction in the central nervous system: relevance to Alzheimer’s disease. *Journal of Alzheimer’s Disease*.

[30] Phiel C. J., Wilson C. A., Lee V. M., Klein P. S. (2003). GSK-3alpha regulates production of Alzheimer’s disease amyloid-beta peptides. *Nature*.

[31] Zhao L., Teter B., Morihara T. (2004). Insulin-degrading enzyme as a downstream target of insulin receptor signaling cascade: implications for Alzheimer’s disease intervention. *Journal of Neuroscience*.

[32] Perez A., Morelli L., Cresto J. C., Castaro E. M. (2000). Degradation of soluble amyloid beta-peptides 1−40, 1−42, and the dutch variant 1−40Q by insulin degrading enzyme from Alzheimer disease and control brains. *Neurochemical Research*.

